# Different Neural Mechanisms Underlie Non-habitual Honesty and Non-habitual Cheating

**DOI:** 10.3389/fnins.2021.610429

**Published:** 2021-02-09

**Authors:** Sebastian P. H. Speer, Ale Smidts, Maarten A. S. Boksem

**Affiliations:** Rotterdam School of Management, Erasmus University Rotterdam, Rotterdam, Netherlands

**Keywords:** dishonesty, cognitive control, multivariate pattern analysis, fMRI, moral default

## Abstract

There is a long-standing debate regarding the cognitive nature of (dis)honesty: Is honesty an automatic response or does it require willpower in the form of cognitive control in order to override an automatic dishonest response. In a recent study (Speer et al., 2020), we proposed a reconciliation of these opposing views by showing that activity in areas associated with cognitive control, particularly the inferior frontal gyrus (IFG), helped dishonest participants to be honest, whereas it enabled cheating for honest participants. These findings suggest that cognitive control is not needed to be honest or dishonest *per se* but that it depends on an individual’s moral default. However, while our findings provided insights into the role of cognitive control in overriding a moral default, they did not reveal whether overriding honest default behavior (non-habitual dishonesty) is the same as overriding dishonest default behavior (non-habitual honesty) at the neural level. This speaks to the question as to whether cognitive control mechanisms are domain-general or may be context specific. To address this, we applied multivariate pattern analysis to compare neural patterns of non-habitual honesty to non-habitual dishonesty. We found that these choices are differently encoded in the IFG, suggesting that engaging cognitive control to follow the norm (that cheating is wrong) fundamentally differs from applying control to violate this norm.

## Introduction

In a recent study (Speer et al., 2020), we found that areas associated with cognitive control, particularly the inferior frontal gyrus (IFG), helped dishonest participants to be honest, whereas it enabled cheating for those who are generally honest. These findings suggest that honest participants needed cognitive control to overcome their inclination for being honest in order to cheat, whereas cheaters had to exert control to override their greedy tendencies in order to be honest. Based on these findings, we argued that cognitive control is not needed to be honest or dishonest *per se* but that it depends on an individual’s moral default.

Our results help reconcile the long-standing debate between proponents of the Will hypothesis and the Grace hypothesis. Research supporting the Will hypothesis (Mead et al., 2009; Gino et al., 2011; Welsh and Ordóñez, 2014) suggests that cognitive control is needed to be honest. In direct opposition to this, a separate stream of research has accumulated evidence in favor of the Grace hypothesis (for meta-analyses, see Greene and Paxton, 2009; Shalvi et al., 2012; Capraro, 2017; Suchotzki et al., 2017; Verschuere et al., 2018), advocating that cognitive control is required for dishonesty. Our findings suggested that people are distributed along a continuum, from individuals who are generally honest to cheaters. Individuals on one end of the continuum are inclined to be honest, which is associated with more self-referential thinking when given the opportunity to cheat. In contrast, individuals on the other end of the spectrum have an inclination for dishonesty, and their decisions are driven more strongly by rewards. In order to achieve a subjectively justifiable and desirable balance where one can occasionally profit from cheating but still maintain a positive self-image, people on both sides of the spectrum sometimes need to override their moral default. We showed that the cognitive control network may orchestrate honesty for people who can be considered cheaters and dishonesty for the more honesty inclined and thus provide potential reconciliation for this long-standing paradox.

In a commentary on our work, Abe (2020) astutely noted that, while our findings provide insights into the role of cognitive control, they do not reveal the exact nature of control-related activity. Although our study demonstrated that activity in the IFG is required when participants override their moral default, our analyses did not reveal whether activity in the IFG for a cheater’s decision to be honest is actually identical to activity in the IFG for an honest person’s decision to cheat. Therefore, the question arises whether, on the neural level, overriding the default to be honest in favor of cheating is identical to overriding the default to be dishonest in favor of honesty. Answering this question would substantially improve our understanding about the nature of the cognitive control processes that enable us to override our moral default, because it may reveal whether the IFG has access to the moral valence (overriding the “good” or “bad” default) of a given decision. To investigate whether neural patterns of activity associated with non-habitual dishonesty can be distinguished from neural patterns underlying non-habitual honesty, Abe suggested the use of multivariate pattern analysis (MVPA, Norman et al., 2006). In this brief report, we apply MVPA to compare neural patterns of non-habitual honesty to non-habitual dishonesty.

## Methods

### Participants

The reported analyses are based on 40 participants (30 females; age 18 to 35 y; M = 23.7, SD = 3.2) recruited from an online community for university students. All participants were right-handed with normal or corrected to normal vision, spoke English fluently, were not on any psychoactive medication influencing cognitive function, and had no record of neurological or psychiatric illness. The study was approved by the Erasmus Research Institute of Management (ERIM) internal review board and was conducted according to the Declaration of Helsinki.

### Spot-the-Difference Task

Participants were presented with pairs of images and were told that there were always three differences between the image pairs (Gai, 2020). Differences consisted of objects that were added to or removed from an image or objects that differed in color between images. However, images could actually contain one, two, or three differences. Participants were asked to find three differences between the images. Because reward (see below) was contingent on participants reporting that they had found all three differences, without having to point them out, this design encouraged cheating behavior (i.e., reporting having found all three, even when objectively fewer than three differences were present in the images).

Participants were told that the purpose of the study was to investigate the underlying neural mechanisms of visual search for marketing purposes such as searching for a product in an assortment or information on a webpage. In order to increase credibility of this cover story a simple visual search task was added at the beginning of the experiment which was also performed in the scanner while participants were undergoing localizer scans.

Further, participants were instructed that the neurocognitive effect of motivation, elicited by monetary reward, on speed and accuracy of visual search was investigated. Although participants were told that there were three differences in all trials, in 25% of the trials, there were only two differences, and in 25% of the trials, there was only one difference. All stimuli were standardized in size and were presented on a white background on a computer screen. The ratio of 50 to 50% (three differences vs. fewer than three differences) was chosen based on the results of pilot studies that indicated this ratio to be optimal in reducing suspicion that the pairs did not always contain three differences.

Trials were further categorized into normal (50%), hard (25%), and very hard trials (25%), for which participants could receive 5, 20, and 40 cents, respectively. All the trials with three differences (the filler trials) were categorized as normal trials, whereas trials with fewer than three differences (the trials of interest) were randomly categorized as hard or very hard trials. Consequently, the reward was independent of the number of differences in the image pair for the trials of interest, which is important in order to be able to disentangle the effects of reward and cheating magnitude (the actual number of differences) on cheating behavior. The different levels of difficulty were added to reduce suspicion about the real purpose of the task. It was assumed that if trials are labeled as hard or very hard, it would be more credible to the participant that the image pair actually contained three differences, but they were just too hard to spot.

In addition, levels of difficulty were introduced to eliminate possible demand effects: we wanted participants to cheat for monetary reward and not to prevent seeming incompetent, which may be associated with different underlying neural mechanisms and consequently confound the analysis. To further reduce suspicion about the purpose of the study, ∼10% of all trials were point-and-click trials. In these trials, participants had to click on the location in the images where they spotted the differences using a joystick. Consequently, cheating was not possible on the point-and-click trials. Participants always knew prior to the start of a trial whether it was a point-and-click trial indicated by a screen requesting participants to click on the image. This ensured that participants would not refrain from cheating on all other trials, while still reducing the suspicion about the real purpose of the study. Participants were told that only 10% of trials were point-and-click trials because it would take too much time to point out the differences for every pair. Further, participants were instructed that excessive movement by manipulating the joystick would interfere with the brain signal. In sum, there were 144 regular trials (of which 72 cheatable trials) and 12 point-and-click trials. The maximum amount of money earned, in case a participant cheated on all cheatable trials, was ∼ €35, whereas in case a participant would not cheat at all, he or she would earn ∼ €7.50.

After completion of the full study, participants were debriefed that the purpose of the study was to investigate the underlying neural mechanisms of (dis) honest decision-making. They were informed that the number of differences between pictures and level of reward were manipulated to encourage cheating. To be fair to all participants, they were all paid out the maximum amount, irrespective of their actual cheating behavior. In addition, participants received a flat fee of €10 for participation in the scanning session.

Each trial started with a fixation cross which was presented for a variable amount of time between 1 and 3 s. Subsequently, the level of difficulty screen was presented for 2 s informing the participants about the level of difficulty of the upcoming trial. This screen also displayed how much money could be earned on that trial. As a result, participants were constantly aware of the potential gains of cheating. Next, an image pair was presented for 6 s, a length determined by the behavioral pilots, and participants engaged in the visual search. Afterward, the participants were asked whether they spotted all three differences (yes/no response). On this decision phase screen, again the potential reward for this trial was presented, in order to make the reward more salient and increase cheating behavior. After 3 s, the response phase started in which participants’ responses were recorded. In the decision phase and the response phase the current balance was also shown, which was done to demonstrate to the participants that if they stated that they had found the three differences, their current balance increased immediately. It was assumed that this direct noticeable effect of behavior on the increase of the current balance would further motivate participants to cheat.

The decision phase and response phase were separated to isolate the decision from motor responses. This was important for the fMRI analysis as we wanted to isolate the neural mechanisms underlying decision-making from possible neural confounds related to button presses. Besides that, the buttons corresponding to “yes” and “no” were switched across trials to further reduce confounding effects and to reduce the response bias for the dominant hand. Once the participants responded, the choice was highlighted by a blue box for 500 ms to indicate that the response was recorded, and the trial ended. If no response was made, the trial ended after 3 s. In addition, there were five practice trials, in which participants could get acquainted with the task. Stimulus presentation and behavioral data acquisition were performed using Presentation software (Version 18.0, Neurobehavioral Systems, Inc.^1^).

### Stimuli

Stimuli for the task consisted of 144 spot-the-difference image pairs that were downloaded from the Internet. Cartoon images of landscapes containing several objects were selected, to make them engaging and challenging enough for the participants. Landscapes were chosen as they generally satisfied the necessary criterion of containing several different objects. The stimuli consist of pairs of images that are identical apart from a certain number (one to three) of differences that were created using Adobe Photoshop. Differences consisted of objects added to or removed from the landscape picture or changed colors of objects. Differences were fully randomized across all pairs of images, which means that all image pairs could be presented with either one, two or three differences. To make sure that participants would be able to find the differences between the images in a reasonable amount of time and to minimize the chance of participants believing that they had seen a difference when they had not (false positives), we ran a pilot study on Amazon’s Mechanical Turk (*n* = 205) to test the difficulty to spot the differences between the images and to determine the optimal duration of picture presentation.

### FMRI Acquisition

The fMRI images were collected using a 3T Siemens Verio MRI system. Functional scans were acquired by a T2^∗^-weighted gradient echo, echo-planar pulse sequence in descending interleaved order (3.0 mm slice thickness, 3.0 × 3.0 mm in-plane resolution, 64 × 64 voxels per slice, flip angle = 75°). TE was 30 ms, and TR was 2,030 ms. A T1-weighted image was acquired for anatomical reference (1.0 × 0.5 × 0.5 mm resolution, 192 sagittal slices, flip angle = 9°, TE = 2.26 ms, TR = 1,900 ms).

### Preprocessing

The fMRI data were preprocessed using fMRIPrep version 1.0.8, a Nipype-based tool (Gorgolewski et al., 2011). The reason for choosing fMRIPrep was that it addresses the challenge of robust and reproducible preprocessing as it automatically adapts a best-in-breed workflow to virtually any dataset, enabling high-quality preprocessing without the need of manual intervention (Esteban et al., 2019). For more details of the pipeline^2^.

### Statistical Analyses

For each participant, we estimated a general linear model (GLM) using regressors for onsets of the decision phase for cheated trials and honest trials. The duration of the epoch for the decision phase was 3 s, and the beginning of the decision phase was used as onset times. The decision phase was used as it provides all the necessary information to make the decision and is free of brain activity related to motor responses. In addition, regressors for the button presses were added. Average background, white matter and cerebrospinal fluid (CSF) signal, framewise displacement, six head motion regressors, and six aCompCor regressors, all obtained from fMRIprep, were entered as regressors of no interest. All regressors were convolved with the canonical hemodynamic response function. A smoothing kernel of 5 mm (FWHW) was applied. Linear contrasts were computed between honest and cheating decisions. Neural patterns were then extracted from the resulting t-maps (cheat > honest & honest > cheat) using the left IFG mask derived from the conjunction analysis between the neurosynth map for cognitive control and the results from the second level analysis investigating the neural mechanisms underlying the decision to cheat in Speer et al. (2020). Due to the fact that participants engaged in spontaneous, voluntary, and deliberate cheating, the ratio of dishonest and honest trials was not perfectly balanced for most of the participants. In order to account for potential statistical confounds resulting from this imbalance, we under-sampled the majority class for each participant to create a perfect balance when estimating the contrasts (Liu et al., 2009).

**FIGURE 1:**
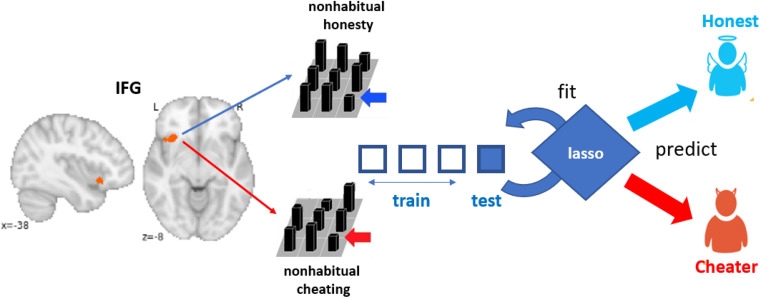
For honest participants, the neural pattern in the IFG was derived from the contrast cheat > honest and for cheaters the neural pattern was obtained from the contrast honest > cheat. These patterns were then fed to a logistic lasso regression classifier, which was trained and tested using 4-fold cross-validation to decode whether a given pattern belonged to a cheater or to an honest participant.

To test whether non-habitual honesty differs from non-habitual dishonesty on the neural level, we conducted a classification analysis on the neural patterns in the IFG (Figure 1) associated with non-habitual honesty in cheaters and non-habitual dishonesty in honest participants. Specifically, we used the t-maps derived from contrasting cheated decisions against honest decisions (cheat > honest) for honest participants and contrasting honest decisions against cheated decisions (honest > cheat) for cheaters as input for the classification analysis. Participants were categorized as cheaters or honest participants based on a median split (median = 10 cheated decisions). The choice of using the honest decision as baseline condition for honest participants and cheated decisions as a baseline for cheaters was motivated by the fact that these decisions represent the default behaviors for the two groups of participants, respectively. We then trained and tested a logistic lasso regression classifier to decode whether a given contrast map represented the neural pattern associated with a non-habitual decision for honesty or dishonesty, using 4-fold cross-validation (Figure 1). The 4-fold cross-validation refers to training the classifier on 30 participants and testing on the 10 remaining participants at each iteration. Importantly, in case the neural patterns associated with non-habitual honesty and dishonesty are the same, the classifier should not be able to accurately categorize cheaters or honest participants based on their neural patterns.

## Results

Substantial individual differences in cheating were observed (mean = 26%, median = 14%, SD = 26%): some participants cheated only once or twice (17.5% of participants), while others only missed one or two chances to cheat (5%). Participants who cheated relatively often are from now on referred to as cheaters and more honest are referred to as honest individuals.

**FIGURE 2:**
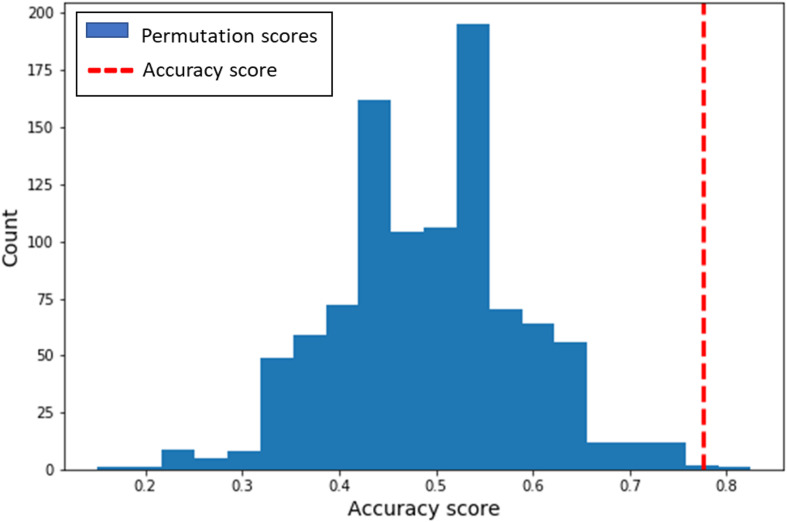
Distribution of classification performance. The blue bars indicate the predictions from the permutation test (*N* = 1000). The red dashed line represents the empirical accuracy score of the model (78%).

**FIGURE 3:**
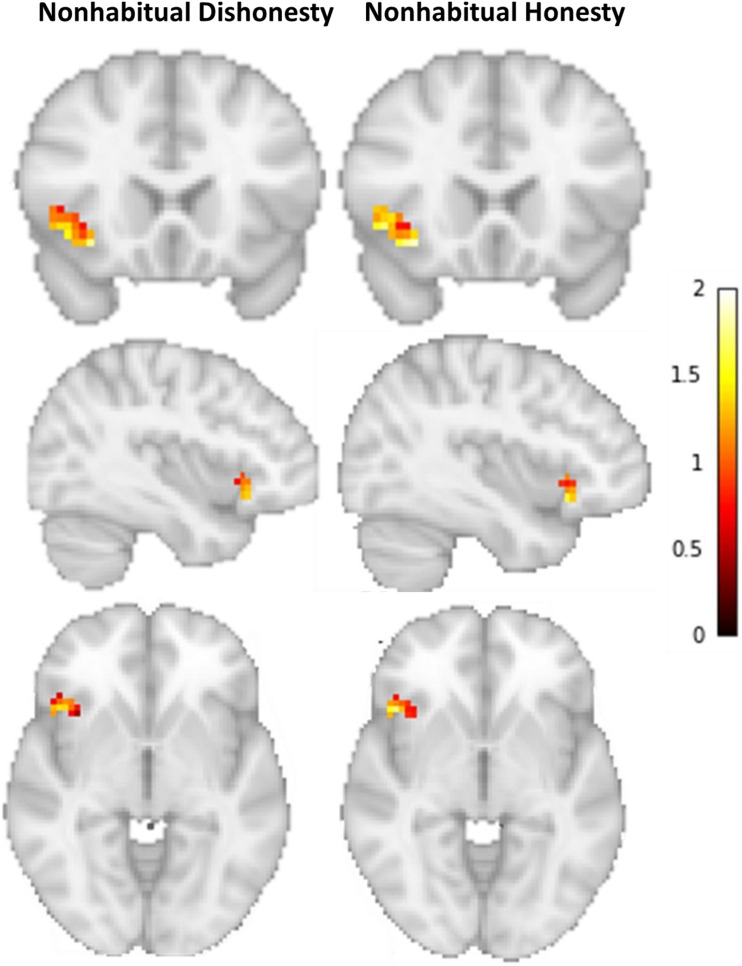
Average activation patterns for non-habitual honesty and non-habitual dishonesty in the left IFG.

The multivariate classification analysis revealed that we can indeed successfully classify whether a participant engaged in either non-habitual honest or non-habitual dishonest behavior (Accuracy = 78%, *p* < 0.01, Npermute = 1000; Cross-validation accuracy scores per fold: 70%, 70%, 80%, 90%; Figure 2). That is, neural patterns of overriding habitual honesty in favor of cheating differ significantly from the patterns underlying a cheater’s decision to be honest. The distribution of activity across voxels within the IFG (Figure 3) suggests that inhibiting habitual honesty (non-habitual dishonesty) is associated within relatively high activity in voxels situated more ventrally in the IFG, while overriding habitual dishonesty (non-habitual honesty) is associated with relatively high activity in more dorsal voxels within the IFG.

In order to make sure that the successful classification can be attributed to differences between non-habitual honesty and non-habitual dishonesty, we ruled out several alternative explanations. First, to test whether the classification accuracy was not just driven by individual differences in honesty irrespective of choice, we trained and tested a logistic lasso regression model to classify cheaters and honest participants using the same contrast for all participants. For both cases, using the honest > cheat and the cheat > honest contrast, the classification accuracy was not significant, indicating that the classification accuracy observed in the previous analysis cannot be attributed to individual differences in honesty alone. Secondly, we explored whether the decoding accuracy mostly resulted from simply using a different contrast (different choices), irrespective of participants’ moral default (individual differences in honesty). To rule this out, we randomly assigned half of the participants to one of the contrasts (honest > cheat) and the other half to the other contrast (cheat > honest) and then used the logistic lasso regression model to decode which participants was assigned to which contrast. The whole procedure was repeated 1000 times. This approach was adopted as it would ensure that the mean level of honesty in the two groups would be the same and thus render the classification of contrasts independent of the participants’ moral default. This analysis resulted in insignificant classification accuracy, which shows that the classification accuracy of the main analysis is not merely driven by the differences in the contrast used.

## Discussion

Using the spot-the-difference task to study trial-by-trial cheating behavior we previously found (Speer et al., 2020) that the effect of cognitive control depends on a participants’ inclination to be honest or dishonest, in other words, on their moral default. The follow-up, analysis presented here revealed that, whereas the level of average activation across all voxels in the IFG is the same for honest participants and cheaters when engaging in a non-habitual (dis)honest decision, the information encoded in the distributed pattern across voxels differs. Specifically, our results hint at differential involvement of dorsal and ventral IFG in non-habitual honesty and dishonesty, respectively.

These results provide deeper insights into the nature of the cognitive control processes that enable us to override our moral default, as they may suggest that the IFG has access to the moral significance of the decision at hand. Engaging cognitive control to follow the norm that cheating is wrong appears to be represented differently in the IFG as compared to applying control to violate this norm. This may suggest that, even though individuals have idiosyncratic default responses in morally ambiguous situations, the underlying moral norm, that cheating is wrong, may nonetheless be universal across individuals. Alternatively, the neural patterns may differ because the specific cognitive processes that need to be inhibited differ. For dishonest participants, the motivation to obtain (monetary) reward needs to be inhibited, while for honest participants the motivation to maintain a positive self-concept needs to be inhibited.

In the field of cognitive neuroscience there has been an enduring interest to refine the constructs of cognitive control (Baddeley and Della Sala, 1996; Cuthbert and Insel, 2013; Diamond, 2013). Particularly, it has been a long-standing challenge to determine which cognitive control processes should be considered domain-general, thus commonly engaged by different types of tasks, and which cognitive control processes are domain or even context specific. The current results provide some initial answers to this question in the context of cognitive control in the form of response inhibition applied to moral decisions. Our findings suggest that, at least in the context of moral choice, inhibiting prepotent responses to cheat are indeed different from inhibiting a default of behaving honestly.

## Data Availability Statement

Data and scripts used for this study are available on Figshare: doi: 10.25397/eur.13625663.

## Ethics Statement

The studies involving human participants were reviewed and approved by the Erasmus Research Institute of Management (ERIM) internal review board. The patients/participants provided their written informed consent to participate in this study.

## Author Contributions

SS, AS, and MB designed the research. SS performed the research and analyzed the data. SS, AS, and MB wrote the manuscript. All authors contributed to the article and approved the submitted version.

## Conflict of Interest

The authors declare that the research was conducted in the absence of any commercial or financial relationships that could be construed as a potential conflict of interest.

## Footnotes

1 www.neurobs.com

2 https://fmriprep.org/en/latest/workflows.html
